# Why Malformations of Cortical Development Cause Epilepsy

**DOI:** 10.3389/fnins.2019.00250

**Published:** 2019-03-29

**Authors:** Alfonso Represa

**Affiliations:** INSERM, Institut de Neurobiologie de la Méditerranée, Aix-Marseille University, Marseille, France

**Keywords:** cortical malformation, epileptogenesis, developmental disorder, ARX, mTOR, focal cortical dysplasia, gray matter heterotopia

## Abstract

Malformations of cortical development (MCDs), a complex family of rare disorders, result from alterations of one or combined developmental steps, including progenitors proliferation, neuronal migration and differentiation. They are an important cause of childhood epilepsy and frequently associate cognitive deficits and behavioral alterations. Though the physiopathological mechanisms of epilepsy in MCD patients remain poorly elucidated, research during the past decade highlighted the contribution of some factors that will be reviewed in this paper and that include: (i) the genes that caused the malformation, that can be responsible for a significant reduction of inhibitory cells (e.g., ARX gene) or be inducing cell-autonomous epileptogenic changes in affected neurons (e.g., mutations on the mTOR pathway); (ii) the alteration of cortical networks development induced by the malformation that will also involve adjacent or distal cortical areas apparently sane so that the epileptogenic focus might be more extended that the malformation or even localized at distance from it; (iii) the normal developmental processes that would influence and determine the onset of epilepsy in MCD patients, particularly precocious in most of the cases.

## Introduction

Malformations of cortical development (MCD) are a complex family of rare disorders that result from alterations of one or combined developmental steps, including proliferation of neural progenitors, migration of neuroblasts to the cortical plate, layer organization and neuronal maturation (Barkovich et al., 2012). Thus, in general, alterations of neuronal and glial proliferation associating neuronal dysgenesis are a cause of focal cortical dysplasia type II (FCD-type II); alterations of neuronal migration leading to ectopic localization of neurons are a cause of periventricular nodular heterotopia (neurons accumulate along the ventricular walls) and subcortical band heterotopia (SBH; neurons accumulate in the white matter between the cortex and the ventricular wall); alterations on processes subsequent to neuronal migration are at the origin of polymicrogyria, characterized by a cortex organized in multiple small gyri (Barkovich et al., 2012).

Malformations of cortical development are an important cause of childhood epilepsy. Though the precise incidence of MCDs is not known, it is estimated that they account for up to 40% of cases of intractable or medication-resistant childhood epilepsies (Barkovich et al., 2012; Guerrini and Dobyns, 2014) and that at least 75% of the patients with MCDs will have epilepsy (Leventer et al., 1999). Associated to seizures, the patients display different comorbidities, particularly cognitive deficits, that are more frequent and severe when epilepsy begins early in life (Berg et al., 2017). MCDs consequently represent a severe burden for patients, families and society.

Though the physiopathological mechanisms of epilepsy in MCD patients remain unclear, clinical and experimental data suggest that epileptogenesis results from diverse developmental processes that can be cell autonomous or not, directly imputable to the genetic cause of the malformation or linked to an abnormal development of neuronal networks. In this review, we will discuss the pathophysiological mechanisms involved in MCD epileptogenesis through the analysis of three distinct matters: the genetic causes of MCDs, the localization of the epileptogenic focus and the age at the epilepsy onset in patients and animal models.

## Causative Genes and Epileptogenesis

The classification of cortical malformations according to the developmental step involved (proliferation, migration or differentiation) or the type of malformation generated (e.g., gray matter heterotopia, lissencephaly, focal cortical dysplasia) does not provide clear indications about the epileptogenic process. However, the analysis of causative genes and their respective cellular roles, provided in some cases interesting data that would help the understanding of the epileptogenic process.

The pathogenesis of MCDs is multifactorial involving different genes and environmental factors. There is a large and increasing number of genes identified during the past decade as causative of MCD (Guerrini and Dobyns, 2014). This is an exciting research field that is also contributing to our knowledge of genetic factors controlling brain development. However, the objective of this report is not to provide a complete overview of the genetic causes of MCDs, but to discuss some examples that help our understanding of epileptogenesis.

One of the more fascinating genes causing brain malformation is ARX (aristaless related homeobox gene). *In vitro* and *in vivo* studies have shown that the Arx gene is both a positive and a negative regulator of gene transcription important for brain development (Collombat et al., 2003; Seufert et al., 2005; McKenzie et al., 2007; Fullenkamp and El-Hodiri, 2008). Among the roles of this gene are the regionalization of the brain, the proliferation of cortical progenitors, the migration of interneurons and early commitment of cholinergic neurons (Colombo et al., 2004; Marsh et al., 2009, 2016; Friocourt and Parnavelas, 2010). Numerous mutations of the ARX gene have been reported in more than a dozen different early neurological disorders, where intellectual disability is associated or not with epileptic seizures (Bienvenu et al., 2002; Kitamura et al., 2002; Stromme et al., 2002). These conditions do or do not associate brain malformations during embryonic development (Shoubridge et al., 2010). Phenotypic heterogeneity may, in part, be explained by the nature and location of ARX mutations (Kato et al., 2004; Olivetti and Noebels, 2012). Indeed, phenotypes without malformation are mainly caused by mutations that are in the polyalanine domains and outside the homeodomain. Conversely, the more severe phenotypes with brain malformation are mostly associated with mutations leading to protein truncation or located in the homeodomain; this is the case for the XLAG syndrome characterized by a severe lissencephaly, agenesis of the corpus callosum and abnormal genitalia. Animal models so far generated have shown that Arx deficient mice or Knockin mice displaying Arx mutations associate a more or less pronounced reduction of cortical GABAergic and cholinergic neurons (reviewed in Olivetti and Noebels, 2012) and the analysis of post-mortem brain tissue reported a three-layered cortex containing exclusively pyramidal neurons in XLAG patients from three different families (Bonneau et al., 2002). In the context of developmental malformations, ARX related syndromes can be thus considered as “interneuronopathies” and the epilepsy and cognitive deficits reported in patients and animal models, would be directly related to the reduction of inhibition. Glutamatergic neurons do not express ARX and are not directly affected by the mutation of ARX but support the consequences and are thus responsible for the expression of epileptic seizures.

Mutations affecting the activation of the mammalian target of rapamycin (mTOR)-signaling pathway have been identified in focal malformations of cortical development associating alterations of progenitor cell proliferation, defective neuronal migration and lamination and the presence of cytomegalic neurons and balloon cells as a result of a defective differentiation program of cortical cells. These malformations include FCD type II and Hemimegalencephaly (Harvey et al., 2008; Blümcke et al., 2011; D’Gama et al., 2017). Tuberous sclerosis, a rare multisystem genetic disease condition that in the brain generates cortical tubers (focal distortions in cellular organization and morphology which extend into the subcortical white matter) is also caused by a hyperactivation of mTORC1, due to mutations in either TSC1 or TSC2 genes (European Chromosome 16 Tuberous Sclerosis Consortium, 1993). This disorder presents intractable epilepsy, cognitive disability, and autism spectrum disorders. Interestingly, tuberal lesion display cellular features similar to FCD type II (i.e., cytomegalic neurons and balloon cells). Therefore, collectively, these disorders might be referred to as “mTORopathies” (Reviewed by Crino, 2015; Marsan and Baulac, 2018). During the past decade somatic activation mutations in mTOR itself have been identified in these syndromes (Lim et al., 2015; Mirzaa et al., 2016; Møller et al., 2016; D’Gama et al., 2017; Ribierre et al., 2018). In addition, positive (e.g., gain of function mutations in Akt1 or AKT3; Lee et al., 2012; Poduri et al., 2012; Jansen et al., 2015) or negative (e.g., TSC2, or DEPDC5; Baulac et al., 2015; D’Gama et al., 2017; Lim et al., 2017) regulators of mTOR have been implicated in FCD type II and Hemimegalencephaly.

Because some patients with FCD are surgically treated, there have been opportunities for investigating on resected tissue neuronal properties (reviewed by Abdijadid et al., 2015). These investigations described for example an abnormal expression of glutamate and GABA receptor in dysplastic and heterotopic neurons (Crino et al., 2001; Lozovaya et al., 2014), a reduction of GABA_A_ -receptor-mediated inhibition (Calcagnotto et al., 2005), an altered pattern of expression and distribution of synaptic protein SV2 (Toering et al., 2009). Carlos Cepeda and coworkers nicely evaluated the electrophysiological properties of cytomegalic neurons and balloon cells and identified in dysplastic areas the presence of neurons with immature cellular and synaptic properties (Cepeda et al., 2007); these observations gave origin to the hypothesis that local interactions of dysmature cells, that would be directly affected by the mutations, with normal postnatal neurons produce seizures (Cepeda et al., 2007). Though these investigations did not elucidate the exact mechanisms of epileptogenesis in FCD, they indicate clearly that different cell types, molecular changes and cellular interactions are contributing factors.

Many neuronal changes reported on neurons from FCD type II or tuber resections would be considered as cell autonomous as they indicate that the hyperactivation of the mTOR pathway leads to neuronal and synaptic dysfunctions, that contribute to epilepsy, independently from the cytoarchitectonic alteration. This notion has been confirmed by Hsieh et al. (2016) when investigating a murine model of type II FCDs by increasing mTOR activity in layer 2/3 neurons of the medial prefrontal cortex. When the hyperactivation of mTOR was induced in cortical progenitors by in utero electroporation, animals displayed dyslamination and cytomegalic neurons; associated to these changes, animals developed spontaneous tonic-clonic seizures. However, when mTOR hyperactivation was induced after corticogenesis, thanks to the use of inducible vectors, animals still displayed an epileptic condition without neuronal misplacement and dysmorphogenesis. In agreement with this, my lab has shown that heterozygous mice from a Tsc1-KO line, develop spontaneous seizures during the first month of life in the absence of any apparent cortical dysplasia (Lozovaya et al., 2014). In this study we demonstrated that Tsc1+/−neurons, particularly L4 spiny stellate cells, display an anomalous expression of NR2C receptors resulting in a change on the kinetics of NMDA currents and that this change was sufficient to induce the epileptic condition. Indeed, the treatment of pups with antagonists specific for NR2C transiently abolished seizures (Lozovaya et al., 2014). We also demonstrated in this report that treatment of newborn mice with rapamycin was sufficient to reverse the phenotype confirming the link between mTORC1 hyperactivation and NMDA receptor changes. Interestingly, cortical resections from patients with tuberous sclerosis and FCD type II also demonstrated higher expression levels of NR2C as compared with control fetal or adult samples (Lozovaya et al., 2014); in addition to this, patch-clamp recordings on these cortical resections demonstrated the contribution of NR2C to NMDA currents confirming the potential role of NR2C to epileptogenesis in mTORpathies.

It is also important to remind, however, that human samples investigated were obtained from severely affected, pharmaco-resistant patients with resection of the epileptogenic zone being the only therapeutic option. The epileptic process by itself can be cause of many subsequent alterations, including an excitatory/inhibitory imbalance. Thus, based on investigations of a Tsc1-KO mice line, Bateup et al. (2013) reported that many biochemical, transcriptional and functional changes in Tsc1 neurons arise secondarily, due to increased network activity. To study the epileptogenic process itself, we must develop appropriate experimental paradigms in order to evaluate the changes that increase the excitability of neurons and networks and that take place before epilepsy onset.

In conclusion, the two paradigmatic examples discussed before, suggest that the initial genetic alteration that yields cytoarchitectonic disruptions of cortical development, might also be responsible for the clinical manifestations. However, these investigations also highlighted to a certain extent the importance of cellular interactions and the possibility that developmental changes, that might involve non-mutated neurons during a particularly vulnerable developmental period, could also contribute to the emergence of epilepsy and/or cognitive deficits.

## The Epileptogenic Network

Investigations combining EEG and functional imaging have demonstrated that patients with focal cortical malformations display interictal or ictal events not only in the affected area (the malformation itself) but also in more or less distal cortical areas. Thus, reports of patients with SBH or periventricular nodular heterotopia indicate that the epileptogenic network is restricted to the heterotopia, or involves both the heterotopia and the surrounding cortex or localizes out of the heterotopia (Mai et al., 2003; Tassi et al., 2005; Kobayashi et al., 2006; Tyvaert et al., 2008; Valton et al., 2008; Christodoulou et al., 2012; Shafi et al., 2015; Pizzo et al., 2017). In patients with FCD, it has also been reported that epileptogenicity extends beyond the limit of the malformation in many patients (Aubert et al., 2009). Another pathological conditions in which the adjacent or even more distal cortex can be the primary origin of epileptiform activity is the tuberous sclerosis. Although tubers are thought to be the initial heart of the epileptogenic zone, electrocorticographic recordings of some patients revealed epileptiform activities and ictal onsets in the perituberal cortex (Madhavan et al., 2007; Major et al., 2009; Ma et al., 2012).

Though conclusions are hampered by the diversities of clinical courses, that includes variabilities on epilepsy onset, type of seizures, efficiency of AEDs, cortical area affected, etc., these data tend to support the notion that epileptic network frequently involves supposedly “healthy” cortical areas that are affected by the presence of a cortical malformation. We investigated this notion in a rat model of SBH induced by in utero (by embryonic day 15) knockdown (KD) of Dcx, the main causative gene of this condition (des Portes et al., 1998; Gleeson et al., 1998; Pilz et al., 1998). In Dcx-KD rats, affected neurons fail to migrate to the cortical plate and form a band of ectopic neurons in the white matter of the electroporated hemisphere (Bai et al., 2003). Dcx-KD rats display altered neocortical excitability already present at the second postnatal week, resulting in an increased propensity for convulsant-induced seizures and spontaneous absence-like seizures in adulthood (Ackman et al., 2009; Manent et al., 2009; Lapray et al., 2010).

We first investigated in Dcx-KD juvenile rats the phenotype of ectopic neurons and found that they displayed a reduced dendritic tree as compared with control (mismatch) neurons and a reduced density of dendritic spines (Ackman et al., 2009; Martineau et al., 2018). We also performed genetic labeling of scaffolding proteins PSD-95 and gephyrin for quantifying, respectively, glutamatergic and GABAergic synapses in ectopic neurons and observed that they were severely reduced (Martineau et al., 2018). These structural changes were associated with a decrease of the frequency of glutamatergic and GABAergic synaptic currents in patch-clamp recordings (Ackman et al., 2009; Martineau et al., 2018). Though DCX also plays a role on neuron maturation (Martineau et al., 2018), the ectopic position of neurons is mainly responsible for the impaired development and synaptogenesis.

Reports on other animal models of cortical migration defects revealed similar features: neurons displayed a simplification of their dendritic arbors in ectopic gray masses induced by either fetal irradiation (Ferrer et al., 1984) or treatment of pregnant rats with methylazoxymethanol (MAM) (Singh, 1980; Chevassus au Louis et al., 1998; Rafiki et al., 1998; Sancini et al., 1998); a significant diminution in spine numbers was also reported in MAM treated offspring (Colciaghi et al., 2014). All together these observations confirm that the positioning of neocortical neurons significantly impacts the subsequent development of dendrites and synapses impairing their integration in functional networks.

In contrast to these dendritic and synaptic defects, ectopic neurons develop axonal projections toward targets that are considered to be “normal” for cortical neurons. In DCX-KD rats, ectopic neurons form cortico-cortical projections like Layer II/III neurons: they reach layers V-VI in the ipsilateral cortex and send axonal projections to the contralateral cortex (Ackman et al., 2009); they also form cortico-striatal connections like Layer V neurons. Similarly, ectopic neurons in irradiated rats and in Tish rats, respectively, lesional and genetic models of SBH, project as normal Layer V neurons to the spinal cord and thalamus (D’Amato and Hicks, 1980; Jensen and Killackey, 1984; Lee et al., 1997). Therefore, ectopic neurons, though poorly innervated as compared with their normotopic counterparts, are in the position of spreading signals to typical cortical targets and participating in the propagation of epileptic activities.

**FIGURE 1:**
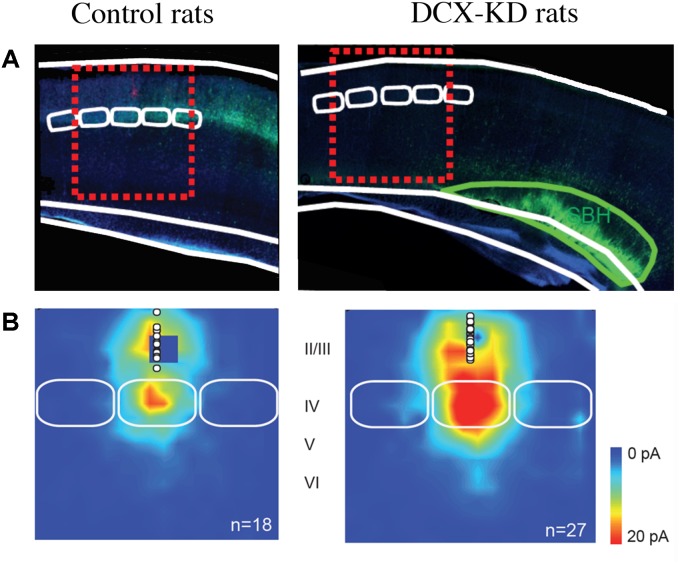
Collateral Alterations of Functional Cortical Circuits in a Rat Model of Subcortical Band Heterotopia. **(A)** Representative examples of across-barrel brain slices from control (Mismatch) and Dcx-KD rats. Fluorescence microphotographs of electroporated cells are superimposed on schematized views of barrels (in white) and SBH (in green). Red dashed line rectangles illustrate the cortical columns evaluated in the study. **(B)** Excitatory synaptic input maps for L2/3 neurons in mismatch and Dcx-KD rats. Colors indicate the mean amplitude of excitatory synaptic responses in a 100-ms time window. White circles show soma positions of recorded L2/3 neurons. Solid white lines delineate barrels. Note that in DCX-KD rats the strength of excitatory inputs to L2/3 neurons are dramatically increased (for more details see Plantier et al., 2018).

We then analyzed the synaptic properties of normotopic neurons from DCX-KD rats to evaluate if the SBH altered the development of normal positioned neurons. Our studies revealed that normotopic neurons displayed an increased frequency of spontaneous glutamatergic post-synaptic currents while GABAergic currents were unaffected (Ackman et al., 2009). As a consequence, the excitatory/inhibitory ratio of synaptic inputs was increased in normotopic neurons (Ackman et al., 2009). Calcium imaging analysis also revealed that more neurons were active in the cortex overlaying SBH than in the cortex of control rats and that they displayed higher frequency of spontaneous events. In addition, more neurons exhibited synchronized events (Ackman et al., 2009). More recently, in a rat model of bilateral double cortex we also observed that the strength of excitatory L4 to L2/3 synapses (Figure 1), the intrinsic properties of L4 glutamatergic cells and the excitation/inhibition ratio in L2/3 converge into making the early stage of cortical sensory integration abnormally strong in somatosensory cortex adjacent to heterotopia, thus demonstrating developmental alterations of cortical functional circuits that likely play a major role in the cortical dysfunction of the malformed brain (Plantier et al., 2018). Investigations of animal models of periventricular heterotopia induced by irradiation (Zhu and Roper, 2000) or treatment with carmustine (Benardete and Kriegstein, 2002) also found an excitatory/inhibitory misbalance in normotopic cortex.

Taken together these data support the notion that normotopic cortex becomes hyper excitable during postnatal development and that it would be responsible for epileptogenesis in DCX-KD rats. Interestingly, the suppression of excitability of ectopic neurons by their transfection with potassium channels Kir2.1, did not alter the high propensity of DCX-KD rats to experience seizures, while the transfection of ectopic and normotopic neurons significantly reduces it (Petit et al., 2014). These observations prompted us to evaluate the origin of interictal events on acute slices from DCX-KD rats using 60-channels microelectrode arrays (Petit et al., 2014). Our data demonstrated that most of interictal discharges initiated in normotopic cortex and propagated secondarily to the SBH. *In vivo* recording with deep electrodes in Tish rats (Chen et al., 2000) also indicated that normotopic neurons were more prone to exhibit epileptiform activities than heterotopic neurons and that blocking the connectivity between the two fields by a local TTX injection inhibited the firing of ectopic but not that of normotopic neurons.

Another pathological condition in which the dysplastic or malformed brain area is not the primary origin of epileptiform activities is provided by the rat model of microgyria induced in rats by freeze-lesioning of deep layer neurons at neonatal stage. Though injured animals did not display spontaneous epileptic events, it has been proven that there is a focal region of hyperexcitability around the lesion (Jacobs et al., 1996); In the cortex lateral to the microgyria, an increased excitation of L5 neurons and an increased inhibition of L5 and L2/3 neurons were observed (Jacobs and Prince, 2005; Brill and Huguenard, 2010; Jin et al., 2014). In addition to this, a rather widespread cortical reduction of GABA_A_ receptor subunits α1, α2, α3, α5, and γ2 expression has been reported (Redecker et al., 2000). These alterations together would account for the increased excitability of an apparently normal cortex lateral to the microgyria.

Collectively, these clinical and experimental observations support the notion that anatomically unaltered cortical regions surrounding the malformation are included in a large epileptogenic network. We propose that developmental changes in these areas play a major role in the generation of more or less large epileptogenic networks.

## The Time of Onset of Epilepsy

The age at epilepsy onset in patients with MCD is largely variable ranging from newborn to adulthood. This is observed even within the same type of cortical malformations. For example, in a series of 132 patients with polymicrogyria (Leventer et al., 2010), the mean age of epilepsy onset was 4.9 ± 6.7 years but ranging from 1 day to 34 years of age. However, 43% of the patients had the first seizures during the first year of life, coinciding with an important period of synaptogenesis. In a similar way, the analysis of 86 female patients with mutations of DCX (Bahi-Buisson et al., 2013) indicate an early onset, during the first year of life, in 55% of cases, but the age at onset varied between the first month of life to 17 years.

These data indicate that the majority of patients with MCD develop the first clinical manifestations of epilepsy during the first year of life, a period of brain development characterized by an intense neuronal maturation and synaptogenesis. It is thus plausible that epilepsy onset is facilitated in these patients by the increasing weigh of maturing synaptic inputs and/or the maturation of the axonal initial segment responsible for the genesis of action potentials and/or other molecular and synaptic changes linked to neuronal maturation. Interestingly, in Dravet syndrome (Dravet, 1978), a severe encephalopathy due to *de novo* loss-of-function mutations in the SCN1A gene, leading to haploinsufficiency of NaV1.1 channel (reviewed by Brunklaus and Zuberi, 2014), epilepsy typically presents around 6 months of age. Expression analysis on human temporal cortex and hippocampus demonstrated that Na(v)1.1 immunoreactivity increased significantly during the late fetal and postnatal periods, reaching peaks 7–9 months after birth (Wang et al., 2011). Hence, epilepsy onset in Dravet patients compares with the developmental course of the affected channel. It is not unlikely that such developmental pattern plays a role on the onset of epileptic manifestations in Dravet and would be also participating in other epileptic syndromes.

It is well established that GABAergic synapses play a major pathophysiological role in epilepsy and thus GABAergic transmission is targeted by many antiepileptic drugs. Synaptic currents induced by the activation of GABA_A_ receptors are carried by chloride and consequently the intracellular concentrations of this anion determine the type of response evoked by the transmitter. In adult neurons, the potassium-chloride cotransporter KCC2 usually extrudes chloride promoting hyperpolarizing, inhibitory, responses. In immature neurons the Na^+^-K^+^-2Cl^−^ cotransporter NKCC1 loads them with chloride and favors depolarizing responses to GABA so that the transmitter might have in developmental brain an excitatory effect (Ben-Ari et al., 1989). However, though the topic remains controversial, the action of GABA on developing cortical neurons *in vivo* seems to be inhibitory (Tyzio et al., 2008; Kirmse et al., 2015; Valeeva et al., 2016), maybe because of its shunting effect (Staley and Mody, 1992) that is independent from the polarity of GABAergic signals. Interestingly, in epileptic tissue from patients with temporal lobe epilepsy it has been shown that changes in chloride homeostasis switch GABAergic signaling from hyperpolarizing to depolarizing (Cohen et al., 2002; Khalilov et al., 2003; Huberfeld et al., 2007). It is therefore likely that GABA may play an important role in childhood epilepsy and that its depolarizing actions in immature neurons would contribute to epilepsy onset. However, in cortical resections from pediatric (6 to 14 months old) Sturge-Weber patients, a severe epileptogenic neurocutaneous syndrome, we found that GABA played mainly an inhibitory and anticonvulsive role (Tyzio et al., 2009). On the other hand, investigations on resections from pediatric patients with FCD, Hemimegalencephaly and tuberous sclerosis (Aronica et al., 2007; Talos et al., 2012) suggest a possible dysregulation on the expression of cation-chloride co-transporters. These changes may be activity-dependent and secondary to the epileptic process itself (Puskarjov et al., 2012); they would thus participate more in the expression and evolution of the epileptic disease than at its onset.

The occurrence of a large time window between the initial insult and the onset of clinical manifestations is a common feature for many neurological disorders, including Parkinson and Alzheimer diseases. In these neurodegenerative diseases the latency window can be related to the evolution of the disease, for example the progressive degeneration of dopaminergic neurons in Parkinson disease. Following this reasoning, the late onset of epilepsy reported in some patients might be due to developmental apoptotic processes and/or synaptic (Bourgeois and Rakic, 1993) and dendritic (Zehr et al., 2006) pruning. It is interesting to note that synaptic pruning is particularly important around puberty. For example, electron microscopy studies in primates (Bourgeois and Rakic, 1993) showed a significant reduction in the density of synapses in cortex and hippocampus, during this period of time and imaging analysis of human cortex depicted a reduction in cortical thickness during equivalent periods (Giedd et al., 2006; Raznahan et al., 2011). Would these changes contribute to the peak of incidence of epilepsy that was observed at this period of life (Doherty et al., 2003)? this including juvenile myoclonic epilepsy, emerging mainly in mid-to-late childhood (Martínez-Juárez et al., 2006; Kasteleijn-Nolst Trenité et al., 2013; Ochoa-Gómez et al., 2017)? We do not have an obvious answer on this, but it is clear that we are facing a particular vulnerable period of brain life and that a deregulation of axonal pruning processes, for example affecting more inhibitory synapses or stabilizing recurrent axonal loops, could reveal at last a neuronal excitability status concealed until then.

Alternatively, the intervention of additional precipitating factors during specific time windows, can be at play. Among the factors that would increase neuronal excitability one can mention hormones like estrogens (Zehr et al., 2006) or progesterone (Smith et al., 2002) and inflammation (Bartolini et al., 2018). There is an increasing interest for the role of inflammation and glia in neurodevelopmental disorders like autism spectrum disorders (ASD), schizophrenia, cerebral palsy, cognitive impairment, epilepsy and depression (Galic et al., 2012; Devinsky et al., 2013; Vezzani, 2013; Knuesel et al., 2014; Marchi et al., 2014; Rosenblat et al., 2014; Jiang et al., 2018). Indeed, microglia could have a significant physiological role, contributing to the regulation of cell death/survival, synapse pruning and neurogenesis (Stolp et al., 2011; Vukovic et al., 2012; Kettenmann et al., 2013; Wake et al., 2013). Microglia thus contribute to the regulation of maturation and plasticity of developing neuronal circuits. Furthermore, it has been suggested that microglia would act as a versatile modulator of neurogenesis depending on its activation state: pro-inflammatory microglia would reduce neurogenesis while anti-inflammatory microglia could increase neurogenesis through release of trophic factors (e.g., Kyritsis et al., 2012) and it is suspected that similar dual action of microglia applies to synaptic functioning, plasticity and stability (Marin and Kipnis, 2013; Nisticò et al., 2017).

The contribution of inflammation and glial cells in epilepsy has been particularly investigated (reviewed by Devinsky et al., 2013; Vezzani, 2013) and it has been proposed that inflammation plays an important role in the onset of pediatric seizures (Bartolini et al., 2018). While some infections (e.g., bacterial meningitis, herpes virus, toxoplasmosis) are known to cause acute seizures (Lowenstein et al., 2014; Vezzani et al., 2016) an actual epileptogenic process would take place in some patients after an initial infection. The precise mechanisms remain to be elucidated but they seem to depend on “*the pathogen itself, the developmental stage, the degree of cytokine-mediated inflammatory response and the genotype-phenotype of the person concerned*” (Vezzani, 2013). It can be thus proposed that in a patient with a susceptible brain condition like MCDs, inflammation-induced responses would act as a second-hit, a trigger or an aggravating factor. Interestingly, signs of activation of both innate and adaptative immunities have been found in dysplastic tissue from FCD type II patients (Iyer et al., 2010), suggesting that at least in this type of malformation an inflammatory process is engaged. To note, however, that some of these changes can be related to the action of mTOR on glial cells or be a consequence of the epileptic activity per se. Future research is required for better understanding this important question.

## Conclusion

**FIGURE 2:**
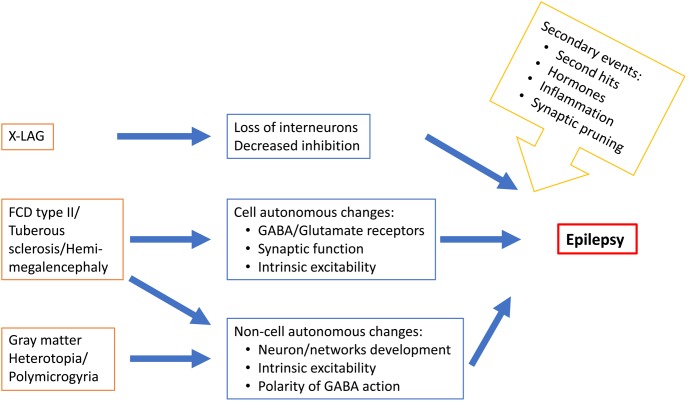
Schematic representation of some MCD-related epileptogenic changes in neurons and neuronal networks.

In conclusion, epileptogenesis in MCDs occur during a period of brain development characterized by many molecular, cellular and structural changes (Figure 2) that determine the features of brain operation and functioning and impact epileptogenic processes and epilepsy expression. Epileptogenesis in MCDs involves complex multifactorial causes that would relate to the type of gene or insult involved in the malformation, normal developmental processes and developmental adaptative or reactive changes in cortical circuitry. The emergence of new animal models reproducing focal mosaic lesions associating manifestation reminiscent of human clinical symptoms, open promising vistas for better understanding the physiopathology of these disorders and testing new therapeutic options.

## Author Contributions

The author confirms being the sole contributor of this work and has approved it for publication.

## Conflict of Interest Statement

The author declares that the research was conducted in the absence of any commercial or financial relationships that could be construed as a potential conflict of interest.
